# Hydroxybenzoic Acids as Acetylcholinesterase Inhibitors: Calorimetric and Docking Simulation Studies

**DOI:** 10.3390/nu14122476

**Published:** 2022-06-15

**Authors:** Grażyna Budryn, Iwona Majak, Joanna Grzelczyk, Dominik Szwajgier, Alejandro Rodríguez-Martínez, Horacio Pérez-Sánchez

**Affiliations:** 1 Institute of Food Technology and Analysis, Faculty of Biotechnology and Food Sciences, Lodz University of Technology, Stefanowskiego 2/22, 90-537 Lodz, Poland; grazyna.budryn@p.lodz.pl (G.B.); iwona.majak@p.lodz.pl (I.M.); 2 Department of Biotechnology, Microbiology and Human Nutrition, University of Life Sciences in Lublin, Skromna 8, 20-704 Lublin, Poland; dominik.szwajgier@up.lublin.pl; 3 Structural Bioinformatics and High-Performance Computing Research Group (BIO-HPC), Computer Engineering Department, Universidad Católica de Murcia (UCAM), Guadalupe, 30107 Murcia, Spain; arodriguez@ucam.edu (A.R.-M.); hperez@ucam.edu (H.P.-S.)

**Keywords:** hydroxybenzoic acids, acetylcholinesterase, Alzheimer disease, ITC, docking simulation

## Abstract

One of the symptoms of Alzheimer’s disease (AD) is low acetylcholine level due to high acetylcholinesterase (AChE) activity. For this reason, AChE inhibitors are used in the treatment of AD, the prolonged use of which may cause a cholinergic crisis. There is a need to search for safe natural AChE inhibitors. The study analyzed 16 hydroxybenzoic acids using calorimetry and docking simulation as AChE inhibitors. All tested compounds were shown to inhibit the hydrolysis of ACh. The best properties were shown by methyl syringinate, which acted as competitive inhibitor at a catalytic site. The tested compounds also interacted with the anionic or peripheral binding site known to block β-amyloid plaques formation. The activity of the tested hydroxybenzoic acids IC50 ranged from 5.50 to 34.19 µmol/µmol of AChE, and the binding constant Ka from 20.53 to 253.16 L/mol, which proves their reversible, non-toxic effect, and activity at physiological concentrations.

## 1. Introduction

Alzheimer’s disease (AD) is the leading cause of dementia in the elderly population. The most important neuropathological changes accompanying AD are the deposition of β-amyloid plaques, the formation of neurofibril tangles, and significant neuronal deficiencies in the brain, resulting in a damaged mechanism of information transmission between synapses [1].

Two mechanisms of the above-mentioned changes are considered: inflammatory and cholinergic [2]. In the case of the latter, the activity of cholinesterases is important, as they hydrolyze acetylcholine (ACh) at a very high rate. Acetylcholinesterase regulates impulse transmission by hydrolyzing ACh at 25,000 particles per second. It is also responsible for interactions with β-amyloid leading to the formation of senile plaques, regulation of cerebral blood flow and phosphorylation of the τ protein [3]. In healthy people, it is responsible for the hydrolysis of excess AChE, and acting together with butyrylcholinesterase, prevents overstimulation caused by high concentration of the neurotransmitter, while in elderly people with ACh low levels, high enzyme activity increases the deficiency of the neurotransmitter and, as a result, causes significant cognitive problems [4]. The pathological condition is exacerbated by the fact that in people with neuronal deficiency, AChE is produced in a higher concentration and is present in large amounts in the form of monomer and dimer, while in healthy people it is mainly tetramer [5]. Therefore, an important therapeutic approach in the treatment of symptomatic AD is the administration of AChE inhibitors, which are the most widely described class of drugs for this neurological disease.

Tarcin, rivastigmine, donepezil and galantamine are clinically available. Synthetic cholinesterase inhibitors, long taken, increase the overall amount of acetylcholine available. Thus, symptoms of overstimulation of the parasympathetic nervous system, such as increased hypermotility, hypersecretion, bradycardia, miosis, diarrhea, and hypotension may occur, with potential development of cholinergic crisis, being a lethal thread [6]. Therefore, the development of new therapeutic AD strategies based on milder therapies is one of the significant topics of research [7].

Many studies indicate the activity of polyphenols as AChE inhibitors, including hydroxybenzoic acids, which, in addition to inhibiting AChE activity, also have an antioxidant effect, including scavenging free radical forms of oxygen, and the ability to chelate transition metals, which reduces the formation of inflammations that can cause the destruction of neuronal structures [8].

The search for natural AChE inhibitors includes research using both plant extracts and single substances. For these purposes, colorimetric and calorimetric methods as well as molecular modelling are used [8]. The latter allow to estimate the activity of a wide range of natural compounds as well as their various synthetic derivatives, however, the most useful seems to be to evaluate the activity of AChE inhibitors, which are phytochemicals found in food. It is known that they are not toxic, and the appropriate composition of the diet of people with AD and possible incorporation into the diet of functional products enriched with natural AChE inhibitors may be the beneficial strategy in the symptomatic therapy of dementia.

AChE belongs to the serine hydrolases. The most important feature of the AChE structure is its wide gorge, half a molecule deep, widening at the base. The active site of the enzyme, located within the gorge, comprises two regions: the anionic choline-binding site and the catalytic esteratic site. The anionic site consists of Trp84, Tyr130, Phe330 and Phe331, and is responsible for binding the quaternary ammonium group of the substrate through cation-π interactions, whereby ACh is properly oriented in the gorge, also known as active cleft. ACh hydrolysis to acetate and choline occurs at the esteratic site. There is a catalytic triad consisting of Ser200, His440 and Glu327. The acyl group of ACh is transferred to Ser200. One of the serine hydrolase activities is to stabilize the substrate transition state by forming hydrogen bonds with the amino acids of the oxyanionic cavity. In AChE, these are Gly118, Gly119, and Ala201. An acyl pocket leads to the active site, which is responsible for the selection of specific substrates. It consists of 14 aromatic amino acids, of which Phe288 and Phe290 are strictly responsible for the shape of the binding pocket and prevent the attachment of larger molecules. The peripheral anionic site (PAS) consists of the five amino acids Tyr70, Asp72, Tyr121, Trp279 and Tyr334, located at the entrance to the active cleft. This is where a number of non-competitive cationic inhibitors attach, which close the possibility of the neurotransmitter from entering the active site and slow down the hydrolysis of ACh. In addition, it is responsible for the binding to β-amyloid and the formation of amyloid plaques [9,10].

Due to the stability of binding with the enzyme, AChE inhibitors are divided into two types: irreversible and reversible. Irreversible inhibitors are highly toxic, e.g., sarin, while reversible inhibitors, both competitive and non-competitive, are of therapeutic importance [11]. The research hypotheses were that some hydroxybenzoic acids have significant activity of reversible acetylcholine inhibitors. The study carried out a calorimetric test (ITC) and docking simulation (DS) showing the activity of AChE inhibitors, which were 16 hydroxybenzoic acids, in order to select the most effective derivatives that could be safely used in the diet of people with dementia.

## 2. Materials and Methods

### 2.1. Chemical Substances

The following compounds of the purity ≥98% were purchased from Sigma-Aldrich (Saint Louis, MO, USA): ethyl 4-hydroxybenzoate (E-4-OH-BA), ethyl vanillate (EV), gallic acid (GA), gentisic acid (GNA), homogentisic acid (HGA), homovanillic acid (HVA), 3-hydroxybenzoic acid (3-OH-BA), 4-hydroxybenzoic acid (4-OH-BA), 4-hydroxyphenylacetic acid (4-OH-PA), 4-hydroxyphenylpyruvic acid (4-OH-PP), nordihydroguaiaretic acid (NDGA), protocatechuic acid (PCA), salicylic acid (SA), sinapic acid (SNA), syringic acid (SRA), vanillic acid (VA), acetylthiocholine iodide (ACh), and Acetylcholinesterase from *Electrophorus electricus* (electric eel) (AChE). Methyl syringate (MS) was purchased from Apin Chemicals Ltd., (Abingdon, UK).

### 2.2. Isothermal Titration Calorimetry (ITC) Analysis

The study was performed to determine protein–ligand interactions using the MicroCal PEAQ-ITC200 apparatus (Malvern, Worcestershire, UK). The analysis was performed according to the procedure of Budryn et al. [12], with some modifications. The measuring cell of the 0.2 mL was filled with degassed AChE solution (1 μmol/L, enzyme diluted with ultrapure water). The syringe of the device was filled with aqueous solutions of hydroxybenzoic acids at a concentration of 0.5 mmol/L or/and ACh at a concentration of 1 mmol/L (neurotransmitter hydrolysis with or without an inhibitor). The solutions were gradually injected into the measuring cell in 2 µL aliquots, where the tested substance was diluted in the enzyme solution and interacted with the enzyme. The analysis was carried out at the temperature of 36.6 °C (mapping the temperature of the human body) with constant stirring (307 rpm). The period between injections was 150 s. During the analysis, the heat absorbed or released as a result of the interactions between AChE and hydroxybenzoic acids and/or ACh was recorded, thus the raw data were collected, obtained as a plot of heat versus time (kcal/s). A total of 19 injections were made within 45 min. Additionally, injection of ligand into water was performed in order to subtract the energetic effects of dilution of the ligand solution.

**Figure 1:**
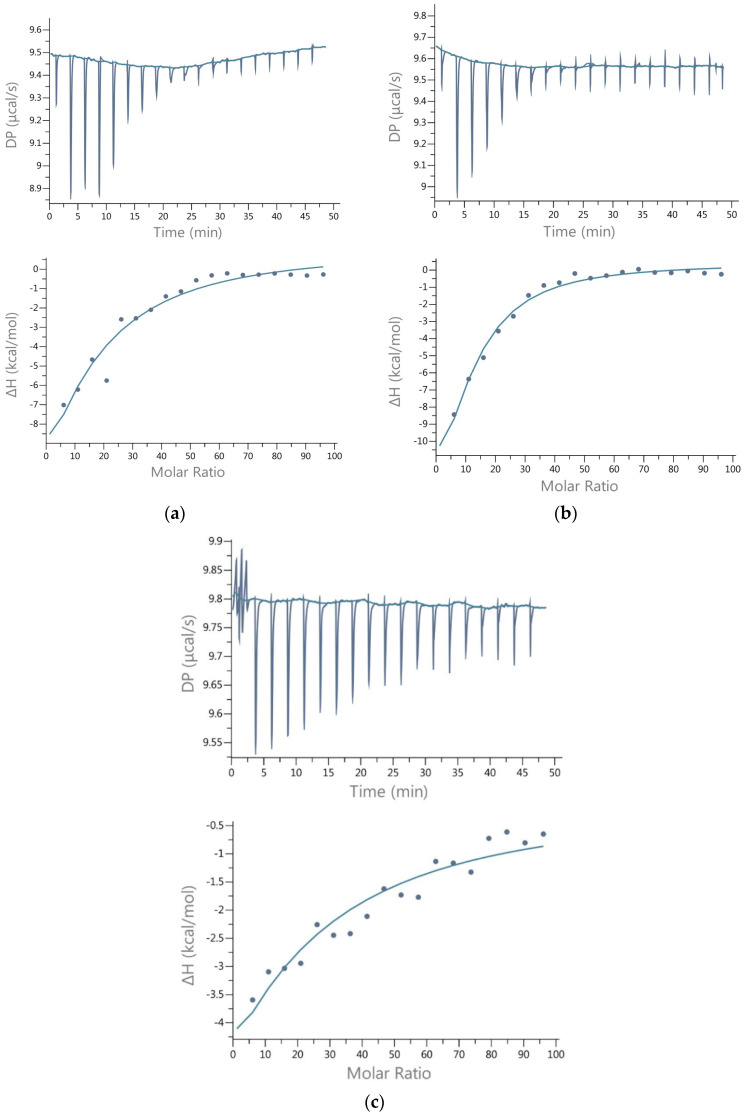
ITC raw data (**a**) ACh hydrolysis catalyzed by AChE; (**b**) ACh hydrolysis catalyzed by AChE in the presence of methyl syringate; (**c**) AChE titrated with methyl syringate.

During the conducted experiments, the complexation (association) constant (Ka) was determined. Data was calculated using MicroCal PEAQ-ITC Analysis software. A “one binding site” interaction model was used. The inhibitory activity was calculated as: [(∆H − ∆Hi)/∆H] × 100%, where ∆Hi is the enthalpy change during ACh hydrolysis in the presence of inhibitor (minus the signal of inhibitor–enzyme interaction and inhibitor dilution), and ∆H is change in enthalpy during ACh hydrolysis without inhibitor. The percent inhibition of ACh hydrolysis at a given molar ligand concentration was calculated and used to calculate the ligand concentration that inhibited the hydrolysis reaction by 50% (IC50). The molar mass of AChE monomer, corresponding to the form occurring in body fluids in pathological states, was assumed in the research. Figure 1 shows examples of recorded energy effects during the ITC analysis.

### 2.3. Molecular Modelling by Docking Simulation (DS)

In order to obtain detailed information at the atomic levels about the interactions between the different hydroxybenzoic acids and AChE, molecular modeling studies were carried out. Docking simulations were performed because their predictions report how electrostatic, van der Waals, hydrogen bonds and hydrophobic interactions between bioactive compounds and AChE are formed [12]. A representative X-ray crystal structure with a code of 1EVE for AChE was taken from the Protein Data Bank (PDB) database (https://www.rcsb.org/structure/1EVE, accessed on 15 November 2021). Next, the full atom model of the enzyme was prepared. Bond orders were assigned, hydrogens were added, and cap termini were included with the Protein Preparation Wizard module available in Maestro [13]. The protonation states of all side chains were subsequently defined using PROPKA3.1. The chemical structures of hydroxybenzoic acids molecules were constructed manually using Gasteiger scheme [14]. The docking of hydroxybenzoic acids to the prepared enzyme structure model was performed with the Autodock Vina docking software [15] using default configuration parameters. The size of the grid box for ligand docking was set to extend 120 Å in each direction from the geometric center of each individual docking simulation. The scoring function from Vina considers the Lennard-Jones term (LJ), hydrogen bonds (H-bonds), electrostatic interactions, hydrophobic stabilization, entropic penalty due to the number of rotatable bonds, and the internal energy of the ligand.

### 2.4. Statistical Analysis

Calorimetric titration was conducted in triplicate. Statistical analysis was based on the determination of the average values of three measurements and their standard deviation, as well as one-way ANOVA (analysis of variation), using Statistica 10.0 software (StatSoft, Kraków, Poland) at the significance level *p* < 0.05.

## 3. Results

The structure of the AChE molecule allows only small molecules to enter the active cleft of the enzyme. Epidemiological observations show that many plant products have anti-neurodegenerative effects that may be associated with blocking AChE. This action may be due to, inter alia, secondary metabolites such as alkaloids, terpenes, coumarins and polyphenols. The latter, including phenolic acids, are commonly found in edible plant foods such as fruits, vegetables, herbs, grains, nuts, and legumes. Among phenolic acids, a significant class of compounds is represented by hydroxybenzoic acids, which are found mainly in berries such as blackberry, blueberry, raspberry, strawberry, black and red currant, in concentrations up to 400 mg/100 g [16]. Their bioavailability is high, the concentration in the blood a few hours after the consumption is even tens μmol/L and may result not only from direct absorption by enterocytes, but also the metabolism of other polyphenols, amino acids or neurotransmitters [17]. 

The studies assessed the inhibition of AChE activity using the ITC and SD interaction model with hydroxybenzoic acids. The calorimetric method, like the colorimetric Ellman’s method, allows the determination of the IC50 value of inhibition of enzyme activity [18], however, in Ellman’s method, the important role of a “false-positive” blank sample in the routine analysis should be considered [19]. The ITC method is a useful tool for verifying the effectiveness of enzyme inhibitors using only a physiological substrate, without additional reagents or secondary reactions. The ITC has the additional advantage that it uses precise heat measurement at physiological concentrations, which is important for the accuracy of the results, because the activity of the inhibitor depends on the concentration of the enzyme and ligand [20,21].

Supplementing calorimetric studies with docking simulation allows to determine the site of complex formation of the enzyme and, as a result, to describe the mechanism of the inhibitor’s action, as well as to design mixtures that, by attaching to different sites of the enzyme, can more effectively block the access of the substrate [3]. Derivatives of hydroxybenzoic acids in the number of 16 were interacted with AChE. Calorimetric titration showed different activity of the tested substances as enzyme inhibitors.

The highest activity, represented as an IC50 of 5.50 μmol/μmol AChE, was shown by methyl syringate (MS), naturally occurring in manuka honey [22], with the binding constant Ka of 154.32 × 10^3^ L/mol (Table 1).

**Figure 2:**
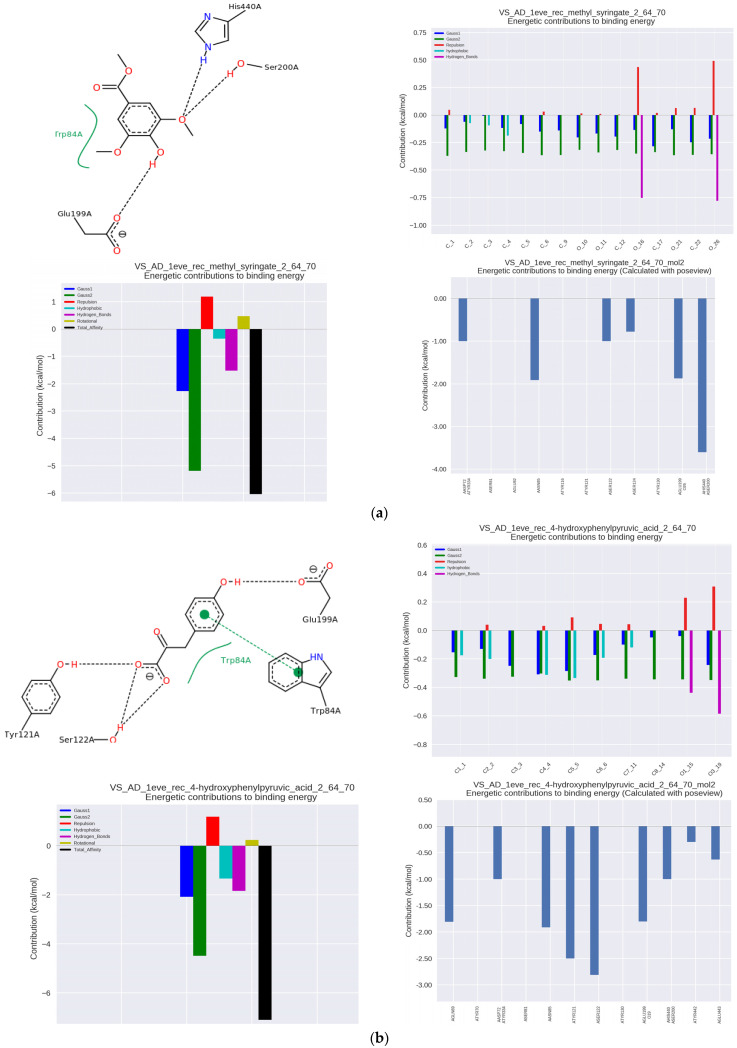

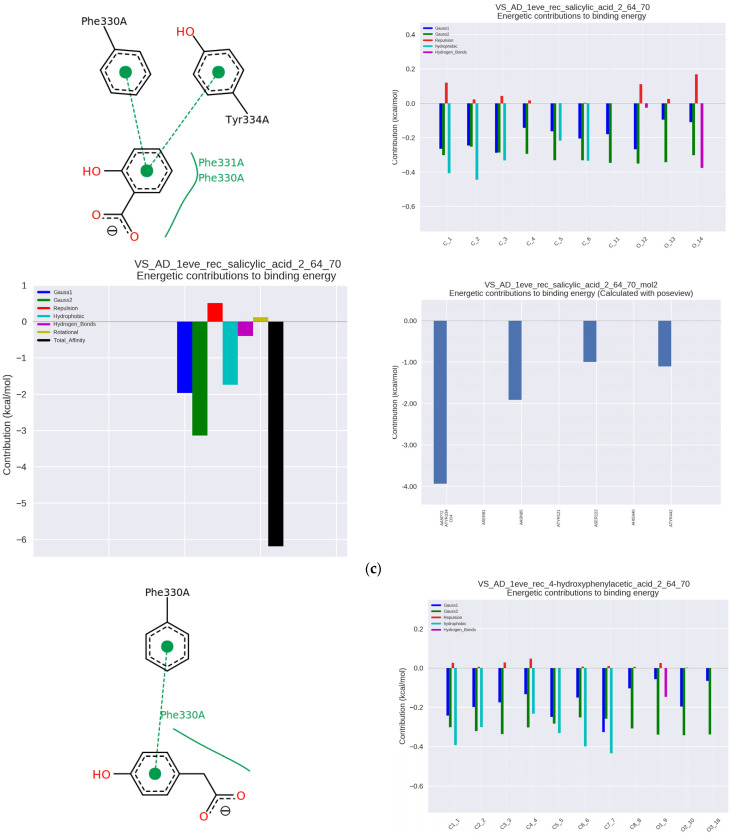

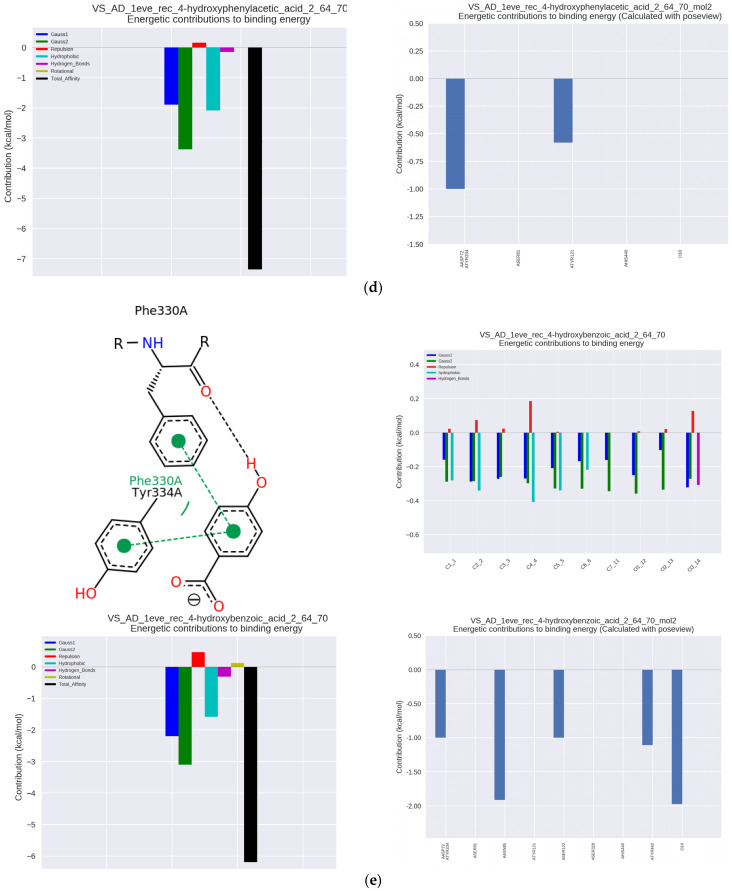

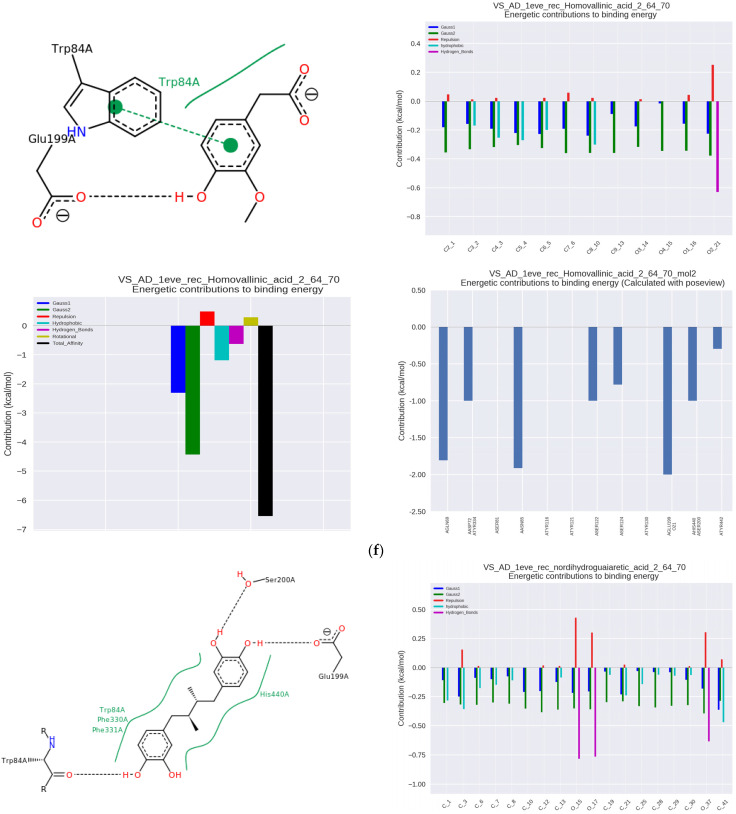

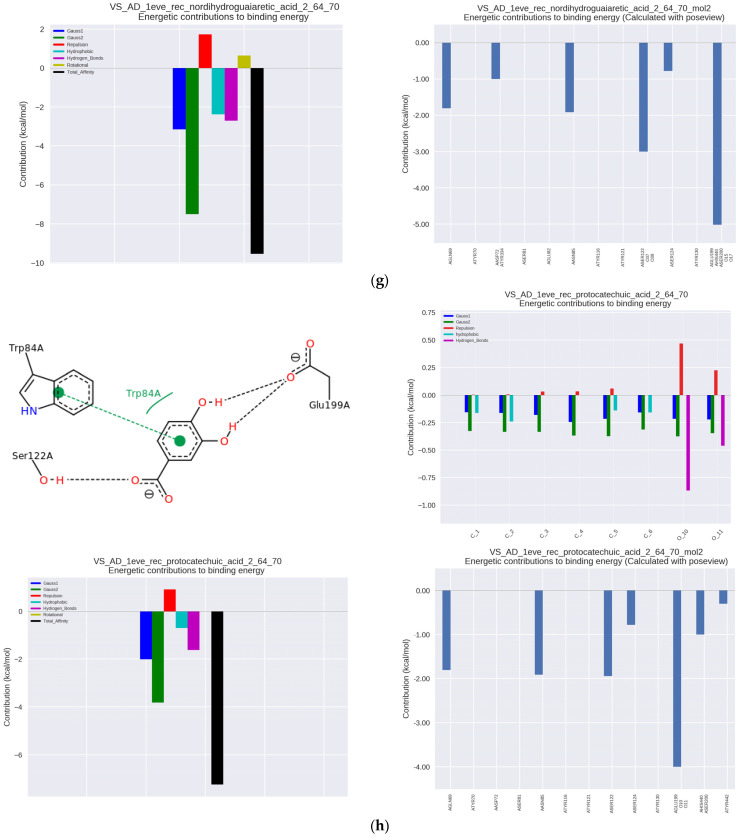

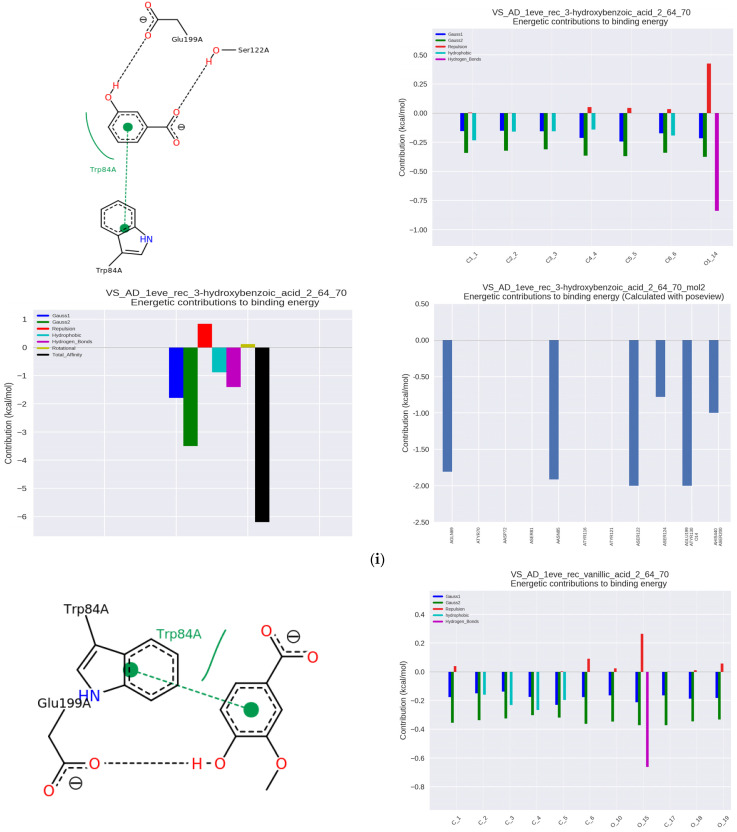

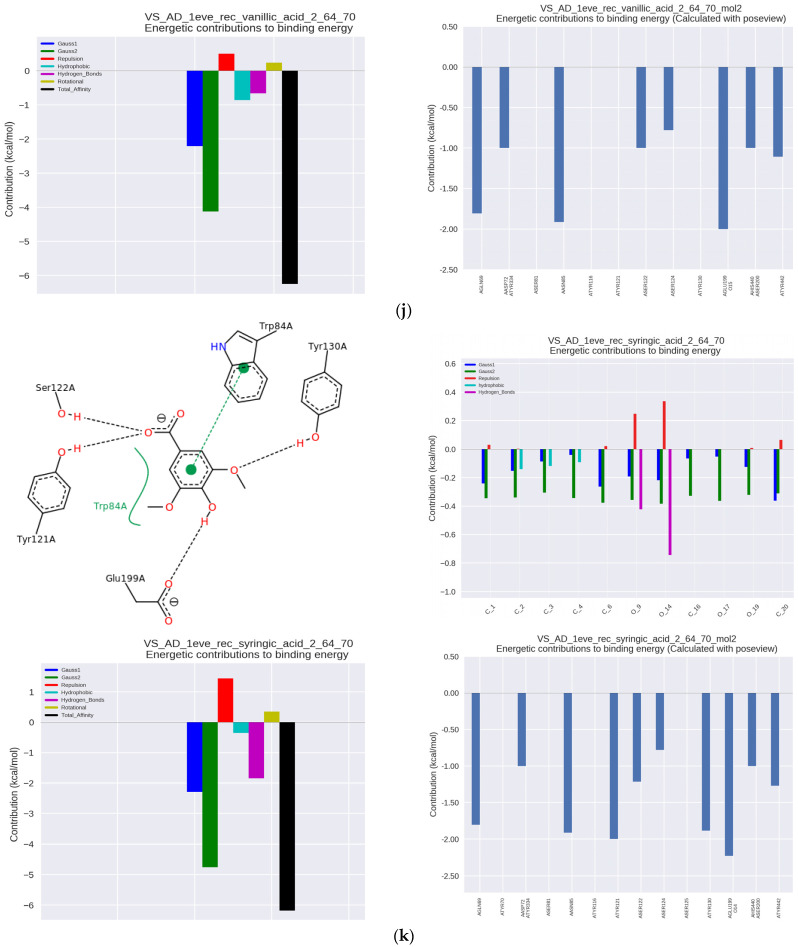

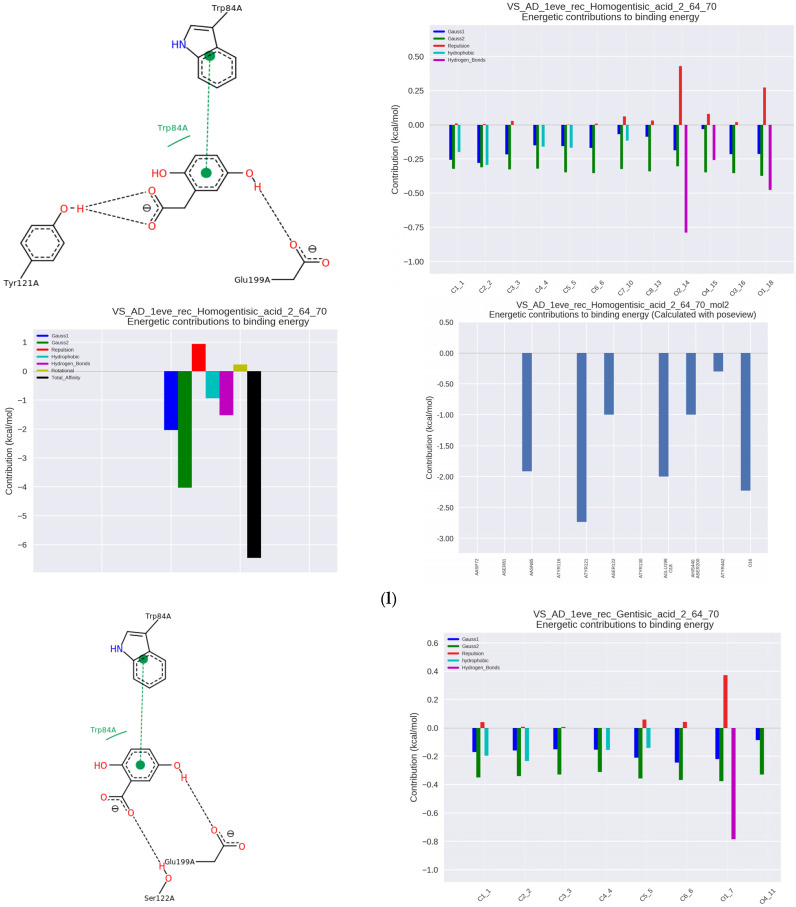

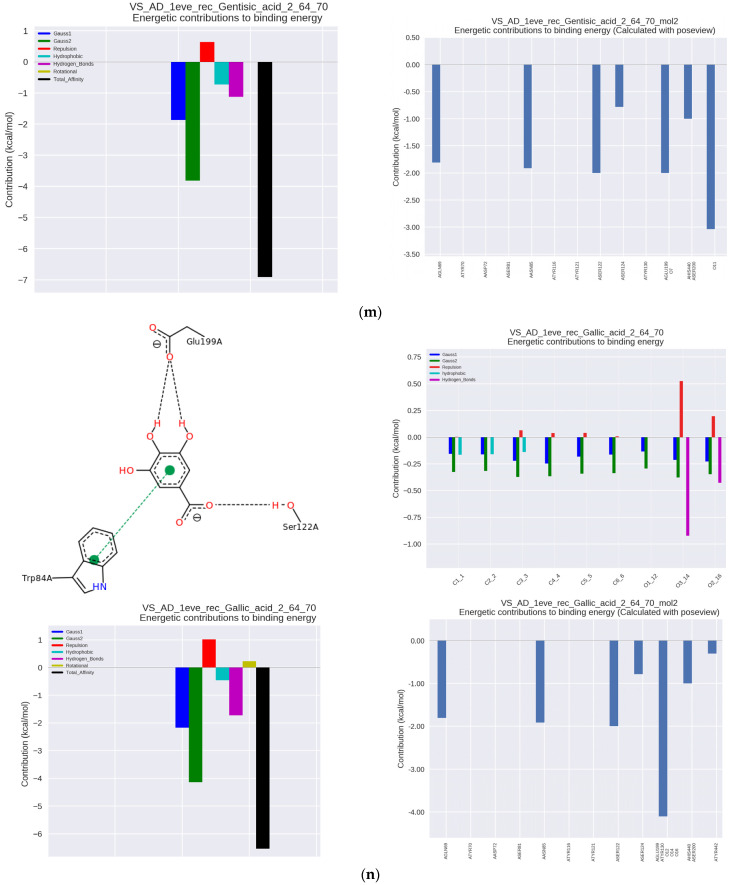

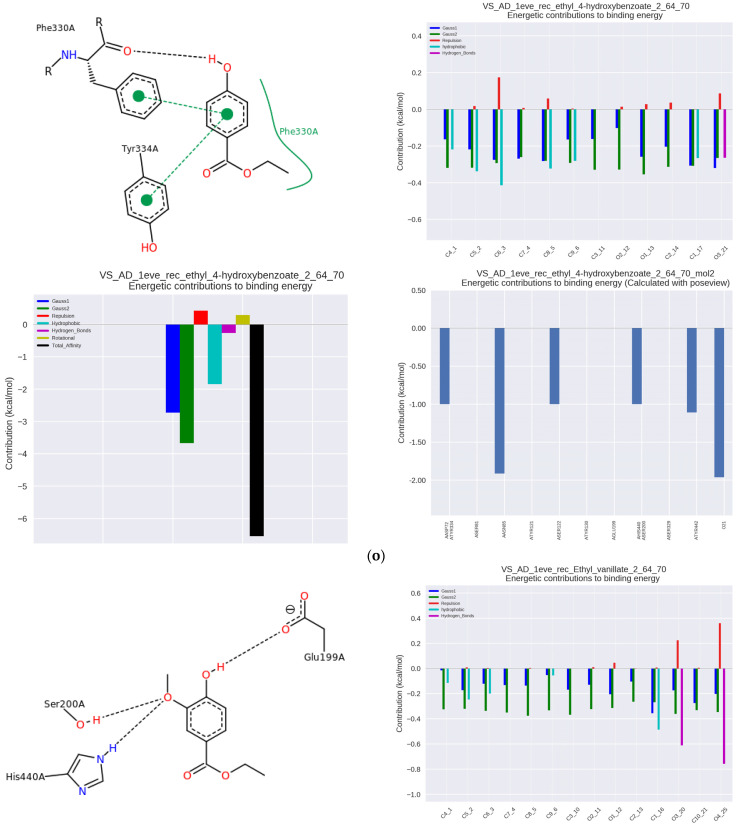

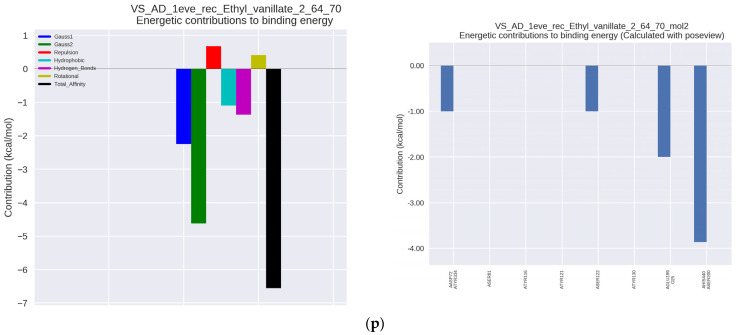
At (**a**–**p**) results of docking simulation. On the top left: a 2D model of the ligand interaction (name in the graph) with AChE; from below: the energy values of the interactions that make up the total binding energy of the ligand with AChE; on the top right: energetic values of the AChE interactions to individual ligand atoms; from below: the total binding energy of the ligand to the most significant interacting AChE amino acid residues.

The nature of the complex with the enzyme included the hydrophobic interaction of the phenolic group with Trp84 and Tyr130 within the anionic binding site (Figure 2a). 

Access to Trp84 is very important for high enzyme activity [11]. The complex was stabilized by hydrogen bonds with the amino acid residues of the Ser200 and His440 within catalytic site, making it a strong competition inhibitor, and additionally with Glu199 in the vicinity of the catalytic site, where the interaction was the strongest and exceeded the energy of hydrophobic interactions. The hydroxyl group binding to the carbonyl oxygen of Glu199 (hydrogen bonding) improves the positioning of the phenyl ring of phenolic acid to involve a π–π stacking interaction with Trp84 [23]. The MS-AChE complex also involved Asp72, Tyr121, and Tyr334 within PAS, the blocking of which prevents aggregation of β-amyloid [26].

ITC analysis of 4-hydroxyphenylpyruvic acid (4-OH-PP), which is a metabolite of some amino acids, showed its very high activity of an AChE inhibitor with an IC50 of 5.89 μmol/μmol of AChE. In complex with the enzyme, hydrophobic and π–π interactions with Trp84 at the anionic binding site were observed, as well as hydrogen bonds with Tyr121 and adjacent Ser122 within PAS (Figure 2b). Additional binding amino acids included Tyr70, Asp72, and Tyr334 in the same region and Tyr130 in the anionic binding site. The complex of 4-OH-PP with the enzyme showed the highest binding constant Ka of 253 × 10^3^ L/mol. The association constant of 105 L/mol is characteristic of complexes with medium binding strength and indicates the reversible nature of the complexation of 4-OH-PP and other analyzed substances.

Salicylic acid (SA) had an IC50 of 6.07 μmol/μmol AChE, and Ka = 51.55 10^3^ L/mol. Hydrophobic interactions with Phe330 and Phe331 in the anionic binding site and π-π with Phe330 and with Tyr334 supported by the binding with Asp72 in PAS were responsible for the high activity. The stability of this complex was determined by the hydrophobic effects, the location of which proves the non-competitive nature of the inhibitor (Figure 2c). SA is found in many foods, including broccoli, cauliflower, cucumber, radish, spinach, zucchini, grapes, pears, peaches, berries, cherries, grapes, plums, pineapples, and other fruits and vegetables [27].

The active inhibitor of AChE was 4-hydroxyphenylacetic acid (4-OH-PA) with an IC50 of 6.24 µmol/µmol AChE, and Ka = 66.23 × 10^3^ L/mol. The hydrophobic and π–π interactions occurred in this case with Phe330 in the anionic active site and determined the predominant share of hydrophobic interactions in the resulting complex (Figure 2d). Weaker interactions were for Tyr70, Asp72, Tyr 121 and Tyr334 in PAS. High concentrations of 4-OH-PA are found in dandelion, evening primrose, wine and beer.

Further, 4-hydroxybenzoic acid (4-OH-BA), which is present in high concentrations in blueberries and coriander, was characterized by high anti-AChE activity. In this case, the IC50 was 6.36 μmol/μmol AChE, and Ka was 58.48 × 10^3^ L/mol, where hydrophobic interactions also predominated, accompanied by hydrogen bonds and π-π with Phe330 in the anionic binding site and additionally π-π with Tyr334 in PAS, where the interactions with Asp72, Tyr121 and Trp334 were also present (Figure 2e).

Another highly active compound was homovanillic acid (HV, IC50 = 6.45 μmol/μmol AchE and Ka = 136.05 × 10^3^ L/mol), a metabolite of dopamine which complex with the enzyme was mainly characterized by π-π and hydrophobic bonds with Trp84 at the anionic binding site, as well as hydrogen binding with Glu199 adjacent to the catalytic site with which 4-OH-BA also interacted weakly via Ser200 and His440 (Figure 2f).

The chaparral plant contains nordihydroguaiaretic acid (NDGA), which was highly active at the IC50 level of 6.47 μmol/μmol AchE and Ka = 57.80 × 10^3^ L/mol. The complex of this compound with AChE involved hydrophobic interactions with Trp84 as well as Phe330 and Phe331 within the anionic binding site, and more importantly, strong hydrophobic interactions with His440, which is located in the catalytic site, and hydrogen one with Ser200 and adjacent Glu199 in the same region, which makes NDGA a competition inhibitor (Figure 2g). The complex was hydrogen-stabilized with Trp84 as well as with Asp72 and Tyr334 in PAS.

Protocatechic acid (PA), found in bitter melon, chicory, buckwheat, black olives or gooseberries, had a very similar anti-AChE activity (IC50 = 6.50 μmol/μmol AchE and Ka = 57.63 × 10^3^ L/mol) despite a slightly different character interactions with hydrophobic interactions at Trp84 and additionally π-π and hydrogen bonds with Ser122 and Glu199, which were important for the stabilization of the complex (Figure 2h). Further analysis of the interactions showed binding to Tyr130, as well as to Ser200 and His440 at the catalytic site characteristic of competition inhibitors. 

Similar effects were shown by 3-hydroxybenzoic acid (BA, IC50 = 6.68 μmol/μmol AchE, Ka = 64.10 × 10^3^ L/mol) found in maize, blueberry, lemon, beer (Figure 2i) and vanillic acid (VA, IC50 = 6.79 μmol/μmol AChE, Ka = 52.36 × 10^3^ L/mol) present, inter alia, in vanilla and Chinese wood, and is also a metabolite of caffeic acid (Figure 2j).

Syringic acid (SA), present, e.g., in olives, dates, herbs, pumpkin, grapes, akai, honey or wine, was characterized by a high binding constant Ka of 183.15 × 10^3^ L/mol, and IC50 = 6.96 μmol/μmol AChE, at the level similar to BA and VA. In this case, the complex was additionally stabilized by hydrogen binding with Tyr121 in PAS, as well as with the adjacent Ser122 and Glu199 next to the catalytic site, which made the hydrogen interactions predominant (Figure 2k).

Another derivative, homogentisic acid (HGA) occurs naturally in Arbutus unedo (strawberry-tree) honey and is also a metabolite of some amino acids. The IC50 was 7.16 µmol/µmol AChE and Ka was 116.92 × 10^3^ L/mol. It attached to AChE at the anionic binding site through hydrophobic and π-π interactions with Trp84 and hydrogen interaction with Tyr121 in PAS (Figure 2l). Additionally, it interacted with the catalytic site Ser200, His440 and the adjacent Glu199.

The related gentisic acid (GNA) is found in grapes, artichokes, avocado, kiwi, sesame, olives, bitter melon, and blackberries. Its anti-AChE activity was IC50 = 8.02 µmol/µmol AChE and Ka = 76.92 × 10^3^ L/mol. Hydrophobic interactions were decisive for the formation of the complex (Figure 2m), and the nature of the interactions was very similar to 3-OH-BA. Similar interactions of the enzyme were with gallic acid (GA, with IC50 of 9.32 μmol/μmol AChE and Ka = 51.55 × 10^3^ L/mol), a very common polyphenol in fruits, vegetables, tea (Figure 2n), as well as with ethyl vanillate, which is a food additive (IC50 34.19 μmol/μmol AChE and Ka = 38.76 × 10^3^ L/mol) (Figure 2o) and with ethylhydroxybenzoate (ethylparaben), used as a preservative (IC50 = 31.38 µmol/µmol of AChE and Ka = 20.53 × 10^3^ L/mol) (Figure 2p). As can be seen, the last two derivatives had significantly lower anti-AChE activity compared to the other tested hydroxybenzoic acids.

## 4. Discussion

The neuroprotective effect of phenolic acids and their closely related derivatives was previously comprehensively elaborated, e.g., in a review by Szwajgier, Borowiec and Pustelniak [24] and Szwajgier, Baranowska-Wójcik and Borowiec [25]. These studies have demonstrated the potential of hydroxybenzoic acids as AChE inhibitors. In the present study, hydroxybenzoic acids were interacted with AChE by the ITC and DS methods, which allowed for obtaining further important information about the activity of these compounds as therapeutics in dementia. The range of 16 compounds of this family were analyzed in the study. These included three isomers of hydroxybenzoic acid, dihydroxy derivatives, homo-derivatives containing an acetic moiety in place of a carboxylic one attached to the phenyl ring. A number of methoxy-substituted derivatives within the carboxyl and phenyl groups were also analyzed. Some of them are abundant in food in both free form, as well as sugar derivatives, mainly glycosides or conjugates with flavanols. These include, but are not limited to, salicylic acid (2-hydroxybenzoic acid), 4-hydroxybenzoic acid, gentisic acid (2,4-dihydroxybenzoic acid), protocatechuic acid (3,4-dihydroxybenzoic acid), gallic acid (3,4,5-trihydroxybenzoic acid) acid) and vanillic acid (3-methoxy-4-hydroxybenzoic acid). Other of the studied derivatives are used as food additives or are characteristic human metabolites of amino acids and other polyphenols.

In the study, 4-hydroxybenzoic acid was compared with the 2-(salicylic acid) and 3-isomer. Of these, salicylic acid showed the lowest IC50, although the binding constant was the lowest among these isomers. 4-Hydroxybenzoic acid showed a slightly lower IC50, and both isomers were characterized by hydrophobic interactions within the anionic binding site and PAS. In contrast, 3-hydroxybenzoic acid interacted with AChE mainly through hydrogen bonds, which shows that the hydrophobic phenol-AChE interactions may be hampered by the specific location of the hydroxyl groups. 

The additional hydroxyl group in the case of protocatechuic acid (3,4-dihydroxybenzoic acid) did not significantly change the binding constant or IC50, but the nature of the interaction with the enzyme changed compared to the 4-hydroxy-derivative, in which hydrogen bonds and this type were of greater importance. The interaction was almost identical to binding of the 3-hydroxy-derivative to AChE, with another hydroxyl group forming an additional hydrogen bond with Glu199, thanks to which the IC50 was statistically lower (*p* < 0.05).

On the other hand, the dihydroxy-isomer: 2,5-dihydroxybenzoic acid (gentisic acid) had significantly lower activity despite the higher binding constant. The presence of the hydroxyl group in position 2 did not cause the formation of a hydrogen bond within this group and allowed for a more hydrophobic nature of interactions compared to the 3,4-isomer. The interactions of gentisic acid with AChE were very similar in nature to 3-hydroxybenzoic acid, and the greater activity of the latter as the enzyme inhibitor could be determined by additional binding to Tyr130 within the anionic binding site.

The presence of three hydroxyl groups in the aromatic ring of gallic acid resulted in a weaker activity of the inhibitor compared to protocatechuic acid with a similar pattern of interaction with the enzyme. In the case of the trihydroxy-derivative, there were interactions that weakened the binding of the complex (lower binding constant) related to the stress of the phenolic acid molecule resulting from the forced partial rotation around the bonds and the greater repulsive force caused by the presence of the hydroxyl group in the hydrophobic interaction region.

The influence of the presence of methyl groups on the interactions of hydroxybenzoic acids with AChE was further analyzed. Vanillic acid (4-hydroxy-3-methoxybenzoic) showed weaker interactions of the hydrogen bond type than protocatechuic acid (dihydroxy-derivative), due to the blocking of the hydroxyl group with a methyl substituent.

In the case of the substitution of two methyl groups in syringic acid, there was a series of hydrogen bonds engaging the oxygen of the SA molecule, not hydrogen, as in the case of the previously discussed derivatives. Hydrogen interactions prevailed in the complex as compared to hydrophobic interactions, and the resulting bonds caused a significant stress on the SA molecule due to repulsion and rotation around the bonds. As a result, the activity of the syringic acid as the enzyme inhibitor was significantly lower than that of gallic acid, a trihydroxy-derivative.

Against this background, the anti-AChE activity of the methyl derivative of syringic acid, methyl syringate, was surprisingly high, but from the picture of the interactions of this phenolic acid with AChE, it can be seen that there are hydrogen interactions with His440 and Ser200, and these amino acid residues are within the catalytic triad and when complexed with the enzyme of this inhibitor, the hydrolysis of the acetylcholine is completely blocked.

Interactions with the enzyme of ethyl derivatives: ethyl vanillate and ethyl-4-hydroxybenzoate were carried out. The interactions were characterized by a low binding constant. Ethyl vanillate showed interactions similar to those of vanillic acid; however, the binding constant was lower in this case, which could translate into a high IC50. Ethyl vanillate interacted with Ser200 and His400 and was a competition inhibitor, however, there were few additional bonds that could stabilize the complex.

In turn, ethyl-4-hydroxybenzoate showed similar interactions with AChE to 4-hydroxybenzoic acid. In the case of the former, higher stresses due to bond rotation, caused by the presence of the ethyl substituent, occurred, and the observed difference in activity was very significant considering the similarity of the interactions. The complexation constant was much lower, so the ethyl group weakened the resulting interactions.

In the last approach, derivatives of homohydroxybenzoic acids were treated with AChE. The activity of 4-hydroxyphenylacetic acid was similar, although slightly higher, to that of 4-hydroxybenzoic acid, but the type of interaction was different. The acetic derivative showed more hydrophobic interactions in both cases at the anionic binding site, and there were practically no repulsive forces. Similarly, homogentisic acid, as compared to gentisic acid, showed a higher activity as the enzyme inhibitor. Homovanillic acid also had a higher activity than vanillic acid, subtle differences in the hydrophobic interactions, greater in the case of the homo-derivative, determined the greater activity of the AChE inhibitor of the latter. 4-Hydroxyphenylpyruvic acid showed a higher activity than 4-hydroxyphenylacetic acid, which could be due to the greater number of significant interactions that stabilized the complex.

An interesting compound was nordihydroguaiaretic acid, which is a benzodiol dimer. Due to the complex structure and the presence of two aromatic groups, it interacted with the enzyme through many amino acids, which could indicate a high energy of complex formation, however, the repulsive forces and stresses resulting from the rotation around the bonds weakened the complex and the activity of this compound was comparable to 4-hydroxybenzoic acid.

Earlier studies by other authors indicated the anti-AChE activity of phenolic acids, including benzoic acids [2,28]. Their structure is similar to some terpenes, which are components of essential oils, which also exhibit the activity of AChE inhibitors [29]. Berberine was very active in in vitro studies of AChE inhibitors, but its toxicity is not precisely defined and human studies of this inhibitor have not been undertaken so far, therefore targeting a diet based on more common nutraceuticals seems to be a better strategy [10].

In our earlier studies carried out using the Ellman`s method, gentisic acid, nordihydroguaiaretic acid, ethyl derivatives and homovanillic acid [23] were characterized by high activity. Some research groups claimed that Ellman’s method does not have sufficient accuracy for measurement of cholinesterases (ChEs) activities. They suggest that when the concentration of the indicator is far higher than the concentration of ACh thiol derivative (ATCh), the hydrolysis rate of ATCh is decreased, resulting in a lower measured ChEs activity.

To some extent, it explains the fact that the results shown in Table 1 indicate a significant dependence of the obtained anti-AChE activity levels of hydroxybenzoic acids and their derivatives on the applied research method. Both the Ellman`s test and the ITC method showed that the level of activity of 16 tested substances did not differ drastically, the differences did not exceed 20 times. Other literature results include, but are not limited to, activity of GA (IC50 = 5.89 μmol/L) [30], of SA (IC50 = 29.53 μg/mL) [31], and vanillin, which is closely related to vanillic acid, was found to be potent inhibitor with IC50 of 37 μmol/L. Remya, Dileep, Tintu, Variyar, and Sadasivan [32] pointed out the role of NDGA as AChE inhibitor with IC50 equal to 46.2 μmol/L. Nugroho, Choi, Hong and Park [33] reported on the anti-AChE activity of SRA (IC50 = 18.73 μg/mL). 

The cited literature data show that the authors use different methodological approaches to investigate the anti-AChE activity of hydroxybenzoic acids, and also other secondary metabolites. This makes it difficult to rationally compare the different works. For this reason, studies that compare several compounds in one study are important.

There are fairly recent studies available on the comparison of the tested compounds to the known AD drugs: rivastigmine, donepezil and galantamine. Acorrding to Yekta, Sadeghi, and Dehghan [34], the kinetic binding parameters of donepezil (60 μM) and AChE (20 mM) was Ka 2.30 × 10^7^ L/mol. The compounds in our study showed a lower association constant, at 10^5^ for: 4-OH-PP (2.53 × 10^5^ L/mol), SRA (1.8 × 10^5^ L/mol) and MS (1.54 × 10^5^ L/mol). On the other hand, studies by Marucci, Buccioni, Ben, Lambertucci, Volpini, and Amenta [35] showed a high inhibitory activity for AChE of donepezil (IC50 = 5.7 nM), rivastigmine (IC50 = 1030 nM) and calantamine (IC50 = 11 nM). This suggests that the available drugs have a lower IC50 compared to the results presented for hydroxybeznzoic acids, at the level of μM. However, looking at the side effects of these drugs, the tested natural substances have the potential for further studies on AChE inhibition.

There is evidence that hydroxybenzoic acids can also exert anti-Alzheimer activity in vivo, although there is little research of this kind compared to protocols involving hydroxycinnamic acids. Vanillic acid reduced (in a dose-dependent manner) AChE activity in mice administered with streptozotocin [36].

The significant anti-AChE potential of this group of compounds is evidenced by the fact that various synthetic derivatives of benzoic acid are used in AChE hydrolysis inhibition studies [37]. Nevertheless, research into the activity of natural derivatives of hydroxybenzoic acid as safe and significant AChE inhibitors should be continued and aimed at indicating active foods to the elderly. What could be the problem? High acidity of fresh fruit, presence of digestive enzyme inhibitors, and possible irritation of the intestine from a diet rich in fresh fruit, resulting in digestive problems. In this situation, the strategy of using fruit processing wastes as a potential source of these compounds seems to be reasonable, accompanied by obtaining concentrates of hydroxybenzoic acids from wastes and including them in functional products friendly to the elderly, e.g., in bread, gruels, soups, etc.

## 5. Conclusions

In this study, 14 derivatives of hydroxybenzonic acids occurring in food and, as metabolites, in humans, as well as two synthetic ethyl derivatives used as food preservatives were analyzed. All of them showed high activity of the acetylcholinesterase inhibitor considering the IC50 value, which showed that at a molar concentration 10 times greater than that of the enzyme, they were sufficiently active, while physiologically their concentrations are even 100- and 1000-times higher than the molar concentration of the enzyme. Against this background, ethyl derivatives substituted in the carboxyl group showed less activity, although they also may have the physiological importance as AChE inhibitors, despite the fact that they are artificial food additives, used in limited concentrations. Hydroxybenzoic acid derivatives attached to the enzyme at various active sites, including the catalytic site characteristic of competition inhibitors. The different binding mode leads to the conclusion that the mixture of hydroxybenzoic acids may be more effective in inhibiting the enzyme activity due to complementary mechanisms: spatial blockade, disruption of proper orientation and ACh binding, and finally the lack of access to the catalytic triad. In the short term, it will now be appropriate to study various plant extracts containing hydroxybenzoic acids selected from the research, which can be incorporated into functional foods. Additionally, research should be continued on the basis of more complex cellular models, enabling the assessment of the protective effect of extracts against the neurodegenerative effects of β-amyloid and, ultimately, in clinical trials. The pharmacokinetic and bioavailability studies of hydroxybenzoic acids are also important.

## Figures and Tables

**Table 1:** Anti-AChE activity studied by ITC method and comparison with colorimetric method results. * Reprinted/adapted with permission from Refs. [23,24,25]. 2022, Dominik Szwajgiwer.

Compound	Abbreviation	Inhibitory Activity			
		ITC			Ellman’s Assay nmol/L of Eserine *
		% Inhibition	IC50 (μmol/μmol AChE)	K_a_ × 10^3^ (L/mol)	
methyl syringate	MS	90.93 ± 7.13	5.50 ± 0.43	154.32 ± 11.12	9.1 ±1.6
4-hydroxyphenylpyruvic acid	x	84.93 ± 5.82	5.89 ± 0.40	253.16 ± 14.53	23.6 ± 1.5
salicylic acid	SA	82.30 ± 4.77	6.07 ± 0.35	51.55 ± 3.77	11.8 ± 0.4
4-hydroxyphenylacetic acid	4-OH-PA	80.20 ± 8.04	6.24 ± 0.63	66.23 ± 5.46	NA
4-hydroxybenzoic acid	4-OH-BA	78.60 ± 8.30	6.36 ± 0.67	58.48 ± 7.81	3.7 ± 0.3
homovanillic acid	HVA	77.50 ± 2.59	6.45 ± 0.22	136.05 ± 5.44	21.8 ± 0.4
nordihydroguaiaretic acid	NDGA	77.20 ± 4.84	6.47 ± 0.41	57.80 ± 4.26	37.3 ± 1.2
protocatechuic acid	PCA	77.15 ± 5.16	6.50 ± 0.43	57.63 ± 2.58	NA
3-hydroxybenzoic acid	3-OH-BA	74.80 ± 8.63	6.68 ± 0.77	64.10 ± 6.04	2.6 ± 0.1
vanillic acid	VA	73.70 ± 1.90	6.79 ± 0.18	52.36 ± 2.53	3.2 ± 0.0
syringic acid	SRA	71.80 ± 3.12	6.96 ± 0.30	183.15 ± 6.69	5.4 ± 0.1
homogentisic acid	HGA	68.78 ± 0.88	7.16 ± 0.09	116.92 ± 2.05	9.6 ± 2.4
gentisic acid	GNA	62.38 ± 6.39	8.02 ± 0.82	76.92 ± 8.78	68.6 ± 0.7
gallic acid	GA	53.70 ± 2.86	9.32 ± 0.50	51.55 ± 3.57	3.8 ± 0.1
ethyl 4-hydroxybenzoate	E-4-OH-BA	15.93 ± 0.75	31.38 ± 1.48	20.53 ± 1.79	28.1 ± 0.1
ethyl vanillate	EV	14.60 ± 2.33	34.19 ± 5.46	38.76 ± 5.91	33.5 ± 2.5

## Data Availability

Not applicable.
